# Workaholism and burnout among *stricto sensu* graduate professors

**DOI:** 10.11606/s1518-8787.2022056003883

**Published:** 2022-05-18

**Authors:** Maynara Fernanda Carvalho Barreto, Maria José Quina Galdino, Frederico Garcia Fernandes, Júlia Trevisan Martins, Maria Helene Palucci Marziale, Maria do Carmo Fernandez Lourenço Haddad

**Affiliations:** I Universidade Estadual do Norte do Paraná Setor de Enfermagem Bandeirantes PR Brasil Universidade Estadual do Norte do Paraná . Setor de Enfermagem . Bandeirantes , PR , Brasil; II Universidade Estadual de Londrina Departamento de Letras Vernáculas e Clássicas Londrina PR Brasil Universidade Estadual de Londrina . Departamento de Letras Vernáculas e Clássicas . Londrina , PR , Brasil; III Universidade Estadual de Londrina Departamento de Enfermagem Londrina PR Brasil Universidade Estadual de Londrina . Departamento de Enfermagem . Londrina , PR , Brasil; IV Universidade de São Paulo Escola de Enfermagem de Ribeirão Preto Ribeirão Preto SP Brasil Universidade de São Paulo . Escola de Enfermagem de Ribeirão Preto . Ribeirão Preto , SP , Brasil

**Keywords:** Faculty, Burnout, Professional, Workload, Occupational Stress, Working Conditions, Mental Health

## Abstract

**OBJECTIVE:**

To analyze the association of excessive work and compulsive work with the dimensions of the burnout syndrome in masters and doctoral professors of Languages, Literature, and Linguistics in Brazil.

**METHODS:**

Cross-sectional study carried out with 585 permanent professors of *stricto sensu* graduate studies in Languages, Literature, and Linguistics in Brazil. Data collection took place between February and August 2019, by an online questionnaire. The outcomes of this study were the compulsive work and excessive work dimensions of the Dutch Work Addiction Scale, the Maslach Burnout Inventory ^TM^ dimensions and their associated factors, identified by multiple logistic regression models.

**RESULTS:**

Professors with a high level of excessive work (29.40%) had 2.75 times the chance of high emotional exhaustion and 2.08 times the chance of high depersonalization. Regarding professors with a high level of compulsive work (8.03%), they had 4.88 times the chance of high emotional exhaustion and 2.97 times the chance of high depersonalization. No association of excessive work and compulsive work with low professional fulfillment was identified.

**CONCLUSION:**

The results showed a statistically significant association of excessive work and compulsive work with high emotional exhaustion and high depersonalization, allowing managers and professors to reflect the criteria that guide their work processes, to adopt management models, institutional regulatory policies, and strategies to improve the working conditions and health of professors.

## INTRODUCTION

Workaholism, a growing phenomenon of addiction or behavioral dependence at work, with progressive development, is characterized by the dimensions called excessive work and compulsive work, drawn from the association of occupational and individual aspects ^1^ .

This phenomenon is among the main causes of negative influence on life and physical and mental illness in workers ^2^ , from an excessive preoccupation with work, stimulated by an uncontrollable motivation, greater time and effort invested in their work activities ^2 , 3^ . Workaholics are individuals who present excessive work behavior and effort dedicated to work compared to the hours of their life ^4^ .

Concerning the development of workaholism in university professors, occupational aspects stand out, in which many hours of dedication are required, with intense mental, physical, and emotional effort to carry out their activities, often characterized by an excessive workload and changes in behavior related to a compulsion to work to reconcile and succeed in their work activities ^5^ .

Workaholism can also harm workers’ interpersonal relationships, influence their health conditions ^6^ , and present itself as a phenomenon positively correlated with other occupational diseases, such as depression and burnout syndrome ^7^ .

Burnout or professional exhaustion occurs from a prolonged response to chronic interpersonal factors and the work environment ^8^ . It is estimated that the syndrome is among the health conditions that affect one in ten workers in different occupational groups ^9^ , with high prevalence among education professionals, such as university professors ^10^ .

Burnout is characterized from three dimensions called emotional exhaustion, depersonalization, and low professional fulfillment ^8^ . Emotional exhaustion refers to the first phase and main factor of the disease, corresponding to a response identified from the reduction of physical and emotional resources of the worker, characterized by the feeling of overload, emotional exhaustion, a feeling of chronic fatigue, lack of energy to performing work activities, excessive irritability, inability to relax and recover homeostatic balance ^8^ .

Depersonalization represents maladjustments related to the interpersonal context of burnout and, when present, workers refer to insensitivity in their interpersonal relationships, excessive detachment from various aspects related to work, adoption of insensitive, impersonal behaviors and attitudes, coldness in work relationships, and reduction in the performance of occupational activities ^8^ .

Low professional fulfillment, also called professional inefficiency, corresponds to the self-assessment component of burnout, since the phenomenon includes cognitive-attitudinal variables such as decreased personal fulfillment at work ^8^ . Regarding the aspects that predict burnout, individual, social, and organizational factors stand out ^11^ .

From the definition of burnout syndrome and workaholism and considering occupational and individual aspects, we highlight the importance of investigating professors who work in graduate programs (PPG – *Programas de Pós-Graduação* ), in which they play a fundamental and strategic role in the production and consolidation of information in the areas of scientific and technological knowledge ^12 , 13^ .

The graduate professors’ work process and their working conditions can result in dissatisfaction and health problems ^14^ , since the activities performed have their own complexity, due to the specificities of the professors’ work process, such as the high workload ^13^ .

We defend the hypothesis that PPG professors are among the professions that are predisposed to develop burnout and workaholism, since there are high demands for scientific production, fragmented activities such as undergraduate classes, external activities associated with graduate studies, among other occupational characteristics that can potentialize the development of these phenomena and their association.

Therefore, knowing the aspects associated with workaholism and burnout and analyzing the association between their dimensions in professors who work in *stricto sensu* graduate programs in Languages, Literature, and Linguistics (PPGLL) is essential, considering that in Brazil studies on burnout in professors, for example, are in a consolidation phase ^15^ .

Given the above, this study presented the research question “Is there an association of excessive work and compulsive work with the dimensions of the burnout syndrome in professors of *stricto sensu* graduate studies in Languages, Literature, and Linguistics in Brazil?”. Thus, this study aimed to analyze the association of excessive work and compulsive work with the dimensions of the burnout syndrome in master’s and doctoral professors of Languages, Literature, and Linguistics in Brazil.

## METHODS

This is a cross-sectional study carried out with professors of master’s and doctoral degrees in Languages, Literature, and Linguistics in Brazil. Permanent professors were included in the PPGLL, totaling 585 participants. Professors who had any type of leave in 2018 were excluded.

To identify the professors belonging to the 155 PPGLL, a manual search was carried out between January and April 2019 on the Sucupira platform, a tool to collect information, carry out analyses and evaluations, and a reference base of the National Graduate System of Brazil ^16^ . For the collection of information regarding the contact emails of each professor, a manual search was carried out on the electronic pages of each program and emails were sent to the course coordinators, requesting information that was not available on their pages, to complement those available on the electronic pages, having identified 2,752 professors accredited to the PPGLL.

Regarding the sample power calculation, the formula n ≥ 50 +8m (m: number of independent variables) was used to test multiple associations ^17^ . Thus, in this study, 38 independent variables were considered, plus the two dimensions of workaholism, which indicated a minimum of 370 participants (n ≥ 50 + (8 x 40)).

Data collection took place between February and August 2019. For this stage, a virtual platform, called Mubble, was developed, containing a semi-structured questionnaire with sociodemographic, health, life and occupational habits variables, in which all questions had mandatory answers.

Burnout syndrome, the outcome of the study, was evaluated by the Maslach Burnout Inventory ^TM^ – Human Services Survey (MBI ^TM^ -HSS) in its version translated into Portuguese ^18^ and validated for use in Brazil ^19^ , consisting of 22 items distributed in its three dimensions: emotional exhaustion (nine items), depersonalization (five items), and low professional fulfillment (eight items). Responses can be measured on a seven-point Likert-type scale, ranging from zero to six.

For the analysis of the answers obtained, the three dimensions must be analyzed and equally considered as a construct, with the objective of considering the syndrome as a potential for development and not as a diagnosis ^20^ . Thus, when individuals simultaneously presents high scores in emotional exhaustion and depersonalization and low scores in professional fulfillment, they may present greater chances for the development of burnout syndrome ^21^ .

Concerning workaholism, the Dutch Work Addiction Scale (DUWAS) was used in its two dimensions called excessive work and compulsive work, translated and validated into Portuguese ^22^ . Therefore, the data were dichotomized into high or low from the 75 ^th^ percentile.

From the database, statistical analyzes were performed with the following variables: sex (male or female), age group (29–49 years old or 50–78 years old), geographic location of the university (Midwest, North, Northeast, South, or Southeast), quality of life (bad or good), leisure opportunities (few or many), sleep pattern (bad or good), physically active (no or yes), type of institution (public or private), two affiliations in PPGLL (no or yes), supervises master’s and doctorate (no or yes), time working in PPGLL (≤ 9 years or ≥ 10 years), number of mentees (≤ 5 students or ≥ 6 students), Capes concept (3–5 or 6–7).

For data analysis, the Statistical Package for the Social Sciences (SPSS), version 20.0 (IBM, Chicago, IL, USA), was used. The outcomes of this study were the emotional exhaustion, depersonalization, and low professional fulfillment dimensions of the MBI ^TM^ -HSS and the excessive work and compulsive work dimensions of the DUWAS, performing bivariate analysis and the calculation of absolute and relative frequencies.

The association between burnout syndrome (dependent variable) and excessive work and compulsive work (independent variables) was verified using multiple logistic regression models, considering the adjustment variables: sex, age group, being a senior professor, and years of experience in the master’s and doctorate. Results were presented as crude and adjusted odds ratios with 95% confidence intervals.

The development of the study complied with national and international research ethics standards, including approval according to Opinion no. 2,347,839, CAAE: 79006017.0.0000.5231.

## RESULTS

Of the 585 participants, most were women (372; 63.59%), and 298 (50.94%) professors were aged between 29 and 49 years old. Regarding health conditions, 423 (72.31%) were not physically active, and 434 (74.19%) considered their quality of life to be good. However, 341 (58.29%) reported having a bad sleep pattern and 459 (78.46%) mentioned not having leisure opportunities.

Concerning the geographic participation of PPGLL, 204 (34.87%) professors were linked to programs in the Southeast region, 165 (28.21%) in the South, 121 (20.68%) in the Northeast, 62 (10.60%) in the Midwest, and 33 (5.64%) in the North.

High emotional exhaustion was associated with age group, quality of life, leisure opportunities, sleep pattern, being physically active, and time working in PPGLL. High depersonalization was associated with age group, geographic location of the university, quality of life, sleep pattern, time working in PPGLL, and Capes concept. Low professional achievement was associated with age group, geographic location of the university, quality of life, leisure opportunities, sleep pattern, time working in PPGLL, and number of mentees ( Table 1 ).

**Table 1:** Association of sociodemographic. occupational. and life habits variables with the dimensions of the burnout syndrome in master’s and doctorate professors in Languages, Literature, and Linguistics in Brazil, 2020.

Analysis variable	High emotional exhaustion		p	High depersonalization		p	Low professional fulfillment		p
	No	Yes		No	Yes		No	Yes	
	n (%)	n (%)		n (%)	n (%)		n (%)	n (%)	
Sex									
Male	112 (52.6)	101 (47.4)	0.77	180 (84.5)	33 (15.5)	0.39	151 (70.9)	62 (29.1)	0.15
Female	191 (51.3)	181 (48.7)		324 (87.1)	48 (12.9)		284 (76.3)	88 (23.7)	
Age range									
29–49 years	126 (42.3)	172 (57.7)	**< 0.01**	244 (81.9)	54 (18.1)	**< 0.01**	209 (70.1)	89 (29.9)	**< 0.01**
50–78 years	177 (61.7)	110 (38.3)		260 (90.6)	27 (9.4)		226 (78.7)	61 (21.3)	
Geographical location of the university									
Midwest	31 (50)	31 (50)	0.19	50 (80.6)	12 (19.4)	**< 0.01**	51 (82.3)	11 (17.7)	**< 0.01**
North	13 (39.4)	20 (60.6)		24 (72.7)	9 (27.3)		20 (60.6)	13 (39.4)	
Northeast	73 (60.3)	48 (39.7)		109 (90.1)	12 (9.9)		101 (83.5)	20 (16.5)	
South	82 (49.7)	83 (50.3)		138 (83.6)	27 (16.4)		111 (67.3)	54 (32.7)	
Southeast	104 (51)	100 (49)		183 (89.7)	21 (10.3)		152 (74.5)	52 (25.5)	
Quality of life									
Bad	39 (25.8)	112 (74.2)	**< 0.01**	121 (80.1)	30 (19.9)	**< 0.01**	92 (60.9)	59 (39.1)	**< 0.01**
Good	264 (60.8)	170 (39.2)		383 (88.2)	51 (11.8)		343 (79)	91 (21)	
Leisure opportunities									
Few	207 (45.1)	252 (54.9)	**< 0.01**	390 (85)	69 (15)	0.10	327 (71.2)	132 (28.8)	**< 0.01**
Many	96 (76.2)	30 (23.8)		114 (90.5)	12 (9.5)		108 (85.7)	18 (14.3)	
Sleep pattern									
Bad	135 (39.6)	206 (60.4)	**< 0.01**	283 (83)	58 (17)	**< 0.01**	235 (68.9)	106 (31.1)	**< 0.01**
Good	168 (68.9)	76 (31.1)		221 (90.6)	23 (9.4)		200 (82)	44 (18)	
Physically active									
No	201 (47.5)	222 (52.5)	**< 0.01**	359 (84.9)	64 (15.1)	0.13	307 (72.6)	116 (27.4)	0.11
Yes	102 (63)	60 (37)		145 (89.5)	17 (10.5)		128 (79)	34 (21)	
Type of institution									
Public	265 (51.4)	251 (48.6)	0.56	445 (86.2)	71 (13.8)	0.87	383 (74.2)	133 (25.8)	0.84
Private	38 (55.1)	31 (44.9)		59 (85.5)	10 (14.5)		52 (75.4)	17 (24.6)	
Two affiliations in PPGLL									
No	235 (50.5)	230 (49.5)	0.23	403 (86.7)	62 (13.3)	0.49	342 (73.5)	123 (26.5)	0.37
Yes	68 (56.7)	52 (43.3)		101 (84.2)	19 (15.8)		93 (77.5)	27 (22.5)	
Advises master’s and doctorate level									
No	88 (47.6)	97 (52.4)	0.16	152 (82.2)	33 (17.8)	0.06	128 (69.2)	57 (30.8)	0.05
Yes	215 (53.8)	185 (46.3)		352 (88)	48 (12)		307 (76.8)	93 (23.3)	
Time working in PPGLL									
≤ 9 years	153 (45.7)	182 (54.3)	**< 0.01**	278 (83)	57 (17)	**< 0.01**	232 (69.3)	103 (30.7)	**< 0.01**
≥ 10 years	150 (60)	100 (40)		226 (90.4)	24 (9.6)		203 (81.2)	47 (18.8)	
Number of advisees									
≤ 05 students	160 (50.5)	157 (49.5)	0.49	272 (85.8)	45 (14.2)	0.79	224 (70.7)	93 (29.3)	**< 0.01**
≥ 06 students	143 (53.4)	125 (46.6)		232 (86.6)	36 (13.4)		211 (78.7)	57 (21.3)	
Capes grade									
3–5	222 (51.3)	211 (48.7)	0.67	364 (84.1)	69 (15.9)	**< 0.01**	321 (74.1)	112 (25.9)	0.83
6–7	81 (53.3)	71 (47.7)		140 (92.1)	12 (7.9)		114 (75)	38 (25)	

PPGLL: *stricto sensu* graduate programs in Languages, Literature, and Linguistics.

Excessive work at a high level was associated with age group, quality of life, leisure opportunities, sleep pattern, being physically active, and time working in PPGLL. High compulsive work, on the other hand, was associated with the variables quality of life, leisure opportunities, sleep pattern, and being physically active ( Table 2 ).

**Table 2:** Association of sociodemographic, occupational, and life habits variables with excessive and compulsive work in master’s and doctorate professors in Languages, Literature, and Linguistics in Brazil. Brazil, 2020.

Analysis variable	Excessive level		p	Compulsive level		p
	Low	High		Low	High	
	n (%)	n (%)		n (%)	n (%)	
Sex						
Male	157 (73.7)	56 (26.3)	0.21	200 (93.9)	13 (6.1)	0.19
Female	256 (68.8)	116 (31.2)		338 (90.9)	34 (9.1)	
Age range						
29–49 years	190 (63.8)	108 (36.2)	**< 0.01**	272 (91.3)	26 (8.7)	0.53
50–78 years	223 (77.7)	64 (22.3)		266 (92.7)	21 (7.3)	
Geographical location of the University						
Midwest	40 (64.5)	22 (35.5)	0.18	56 (90.3)	6 (9.7)	0.96
North	22 (66.7)	11 (33.3)		32 (97)	1 (3)	
Northeast	89 (73.6)	32 (26.4)		111 (91.7)	10 (8.3)	
South	108 (65.5)	57 (34.5)		151 (91.5)	14 (8.5)	
Southeast	154 (75.5)	50 (24.5)		188 (92.2)	16 (7.8)	
Quality of life						
Bad	84 (55.6)	67 (44.4)	**< 0.01**	124 (82.1)	27 (17.9)	**< 0.01**
Good	329 (75.8)	105 (24.2)		414 (95.4)	20 (4.6)	
Leisure opportunities						
Few	308 (67.1)	151 (32.9)	**< 0.01**	414 (90.2)	45 (9.8)	**< 0.01**
Many	105 (83.3)	21 (16.7)		124 (98.4)	2 (1.6)	
Sleep pattern						
Bad	214 (62.8)	127 (37.2)	**< 0.01**	301 (88.3)	40 (11.7)	**< 0.01**
Good	199 (81.6)	45 (18.4)		237 (97.1)	7 (2.9)	
Physically active						
No	284 (67.1)	139 (32.9)	**< 0.01**	383 (90.5)	40 (9.5)	**< 0.01**
Yes	129 (79.6)	33 (20.4)		155 (95.7)	7 (4.3)	
Type of institution						
Public	362 (70.2)	154 (29.8)	0.52	475 (92.1)	41 (7.9)	0.83
Private	51 (73.9)	18 (26.1)		63 (91.3)	6 (8.7)	
Two affiliations in PPGLL						
No	325 (69.9)	140 (30.1)	0.46	429 (92.3)	36 (7.7)	0.61
Yes	88 (73.3)	32 (26.7)		109 (90.8)	11 (9.2)	
Advises master’s and doctorate level						
No	126 (68.1)	59 (31.9)	0.37	172 (93)	13 (7)	0.54
Yes	287 (71.8)	113 (28.2)		366 (91.5)	34 (8.5)	
Time working in PPGLL						
≤ 9 years	221 (66)	114 (34)	**< 0.01**	307 (91.6)	28 (8.4)	0.73
≥ 10 years	192 (76.8)	58 (23.2)		231 (92.4)	19 (7.6)	
Number of advisees						
≤ 5 students	222 (70)	95 (30)	0.74	292 (92.1)	25 (7.9)	0.89
≥ 6 students	191 (71.3)	77 (28.7)		246 (91.8)	22 (8.2)	
Capes grade						
3–5	302 (69.7)	131 (30.3)	0.44	399 (92.1)	34 (7.9)	0.79
6–7	111 (73)	41 (27)		139 (91.4)	13 (8.6)	

PPGLL: *stricto sensu* graduate programs in Languages, Literature, and Linguistics.

Table 3 shows that professors with a high level of excessive work had 2.75 times the chance of high emotional exhaustion and 2.08 times the chance of high depersonalization. Professors who showed a high level of compulsive work had 4.88 times the chance of emotional exhaustion and 2.97 times the chance of high depersonalization.

**Table 3:** Association between the dimensions of burnout syndrome and workaholism in professors of master’s and doctorate degrees in Languages, Literature, and Linguistics in Brazil. Brazil, 2020.

Multiple model	p	Odds ratio ^Crude^ (95%CI)	p	Odds ratio ^Adjusted^ (95%CI)
High emotional exhaustion				
Excessive work	**< 0.01**	2,082 (2,119–4,483)	**< 0.01**	2,754 (1,876–4,043)
Compulsive work	**< 0.01**	5,087 (2,412–10,729)	**< 0.01**	4,876 (2,291–10,377)
High depersonalization				
Excessive work	**< 0.01**	2,298 (1,423–3,713)	**< 0.01**	2,076 (1,272–3,387)
Compulsive work	**< 0.01**	2,982 (1,518–5,860)	**< 0.01**	2,972 (1,495–5,907)
Low professional fulfillment				
Excessive work	0.07	1,451 (0,977–2,155)	0.14	1,356 (0,904–2,034)
Compulsive work	0.74	1,119 (0,574–2,183)	0.73	1,127 (0,573–2,218)

95%CI: 95% confidence interval.

Note: adjusted by sex, age range, senior professor and years working in master’s and doctorate teaching.

## DISCUSSION

The analysis of the association of excessive work and compulsive work with the dimensions emotional exhaustion, depersonalization, and low professional fulfillment of burnout syndrome showed that professors with high levels of excessive work and compulsive work had increased chances of emotional exhaustion and depersonalization. It was observed that excessive work and compulsive work were not significantly associated with low professional fulfillment.

A study by Clark et al. ^23^ found, by meta-analysis, a positive relationship between workaholism and burnout. The study results showed greater correlations with the dimensions of emotional exhaustion (p = 0.42) and depersonalization (p = 0.29) compared to low professional fulfillment. Another study ^4^ , carried out to compare workaholism as a predictor for burnout in occupational groups in China and the United States, showed workaholism positively correlated with emotional exhaustion and depersonalization. The results of both investigations corroborate the findings of this study.

Regarding teaching work, especially in master’s and doctorate courses, the intensification of work activities can be a factor that potentiates excessive work, compulsive work, and, consequently, emotional exhaustion and depersonalization. This factor refers to the increase in a worker’s expected workload, increasing the number of tasks to be performed or decreasing the time needed to complete the required demands ^4^ .

It is assumed that teaching work results in an intense cognitive strain in which professors face more than 40 hours of work per week, in addition to making time to take part in extracurricular activities, bureaucratic activities, and being involved in the development of research. Thus, with an increase in delegated functions and activities, workers spend more hours to meet their deadlines and functions, so that more working hours can lead to greater fatigue, work stress, and professional life imbalance ^4^ .

An important study ^24^ carried out with university professors from 13 countries showed that, in 2012, the average hours worked by its participants was 48.4 hours per week, and about 20% of them reported working more than 60 hours between Monday and Friday.

Regarding the variables that can be associated with both workaholism and burnout in university professors, especially those working in master’s and doctorate courses, the pressure for the development of research and scientific publications are the main factors of occupational stress ^14^ and may be associated with high levels of emotional exhaustion and depersonalization ^25^ .

It is noteworthy that emotional exhaustion increases as workers have a greater perception that the profession is stressful, believe that it is interfering with personal life, and/or consider their work activities less interesting than when they started. Concerning depersonalization, its levels can increase with longer service time, perception of family expectations, poor student behavior, lack of autonomy, stability and work as a total living space, and lower satisfaction with professional growth ^16^ .

As for the factors associated with the dimensions of workaholism and burnout in this study, the percentage of professors aged up to 49 years old with high emotional exhaustion, high depersonalization, low professional fulfillment, and high level of excessive work was higher than among professors over 50 years of age. These findings corroborate the assertion that workaholism may be significantly correlated with the age group in which younger individuals present high levels of excessive work and compulsive work compared to older individuals ^26^ .

It is noteworthy that burnout is common among younger professors ^27^ . Regarding its dimensions, emotional exhaustion decreases with increasing age and depersonalization can increase with increasing age, being more common in the age group from 40 to 59 years old. However, results related to the relationship between these variables, in professors, are still incipient ^15^ , especially for professors who work in PPG.

Concerning geographic location, there was a significant difference between the region of the university in which the participants worked for depersonalization and low professional fulfillment. This may be related to the inequality in the construction and regional distribution of PPGs in Brazil, with a strong concentration in the richest regions of the country ^13^ , favoring the Southeast, South, and Federal District regions, mainly the states of Rio de Janeiro and São Paulo ^28^ .

We defend the hypothesis that the asymmetry and inequalities arising from the construction of *stricto sensu* graduate studies in Brazil have an influence on the profile of PPGLL, program requirements in the development of teaching work, the way in which professors feel and perform their functions, and, thus, the working, physical, and mental health conditions of professors.

The professors in this study who reported having worked in PPGLL for more than ten years had lower percentages of high excessive work, high emotional exhaustion, high depersonalization, and low professional fulfillment compared to professors with less than nine years of experience. However, the percentage of low professional fulfillment was lower among professors who claimed to have more than six mentees.

It is noteworthy that no studies were identified to support the discussion of these results; nevertheless, it is assumed that professors who have more than six mentees have a longer teaching time, present consolidated careers and a greater coping strategy regarding occupational aspects of their work processes, contributing to greater professional satisfaction and pleasure at work.

Professors belonging to courses with Capes concept six and seven showed a lower percentage of high depersonalization compared to courses with grades between three and five. Although the characteristics of the work are different in private and public institutions ^14^ , the assessment requirements regarding PPGs by the Coordination for the Improvement of Higher Education Personnel (Capes), a government agency that regulates graduate studies in Brazil, are unique for the area.

However, it is assumed that PPGLL with concepts from three to five are in search of consolidation and excellence, as well as of recognition of their programs, maintenance and expansion of resources for the courses, and, based on the requirements presented to professors and the evaluation model adopted, in the long term, these may be factors associated with the depersonalization of these professionals.

Concerning the evaluation model of *stricto sensu* PPG carried out by Capes, the competition that emerges from the requirements imposed for the performance of master’s and doctorate courses, permanence or withdrawal from these programs, are factors of questioning on the part of professors regarding their sense of belonging to the programs. In the case of the Languages, Literature, and Linguistics area, despite the existence of metrics for evaluating the area, there is also the exclusion of humanities in public funding policies and its historical constitution marked by the lack of support from funding agencies, the destination of less resources and incentives for research compared to the areas that constitute the Human Sciences, and the need to fight productivism in graduate studies and its effects on Brazilian scientific policy ^29^ .

Still on productivism, demanding productivity and obtaining financial resources for research is associated with a cyclical process in which the subjective perception of pressure for publications and products can imply an increase in the pace and workload and, as a consequence, in excessive commitment, stress, and greater effort to develop work activities ^14^ .

The professors in this study who claimed to have a good quality of life had a lower percentage of high levels of excessive work and compulsive work, high emotional exhaustion, high depersonalization, and low professional fulfillment, compared to professors who reported having a poor quality of life.

The quality of life of an individual constitutes an important health factor that can be affected by workaholism, because, from the behavior of excessive and compulsive work, worker fatigue is often not recognized ^3^ . Regardless of the culture, workaholism is harmful to the well-being of workers because it is associated with high levels of tension at work and, thus, high levels of emotional exhaustion, low quality of life, and compromised sleep quality ^26^ . Therefore, both individuals and work organizations need change strategies ^4^ .

In this study, participants who reported having a good sleep pattern had a lower percentage of high levels of excessive work and compulsive work, high emotional exhaustion, high depersonalization, and low professional fulfillment, compared to professors who reported having a bad sleep pattern. We also add that the professors in this study who reported being physically active had a lower percentage of high excessive work, high compulsive work, and high emotional exhaustion. Physical activity and sleep are important for human recovery, minimizing irritation or exhaustion in the body and mind ^5^ .

Regarding physical activity, the World Health Organization recommends that people between 18 and 64 years of age practice at least 150 minutes of light or moderate activity weekly, or at least 75 minutes of higher intensity activity ^30^ . This practice is considered a protective factor for burnout in university professors ^10^ . Nevertheless, a study ^5^ carried out with 270 university professors showed a lack of physical activity in their free time by almost a third (29.6%) of the participants, similar to the findings in this study.

As for sleep, it is considered an important factor of protection, physical restoration, and health balance for human beings, as long as it is sufficient and of good quality. However, imbalances in this factor can affect the individual’s cognitive, mental, and physical performance and are considered a serious problem in maintaining the health of workers, especially workaholics ^26^ .

Imbalances in the pattern and quality of sleep are considered factors that constitute one of the results of the worker who is addicted to work in different occupational groups, resulting in a lower quality of life ^2 , 26^ . University professors have a significant association between excessive daytime sleepiness and lower scores on items such as functional capacity, physical aspects, pain, general health status, vitality, social aspects, emotional aspects, and emotional health ^5^ .

The professors in this study who reported having many leisure opportunities presented a lower percentage of high levels of excessive work and compulsive work, high emotional exhaustion, and low professional fulfillment, compared to professors who mentioned having few leisure opportunities. From this perspective, workers with high levels of excessive work and compulsive work end up investing their cognitive, emotional, and physical resources in the work process, but are not always able to replenish their resources by restorative activities such as leisure ^4^ .

In addition, the use of technologies and means of communication available 24 hours a day, commonly used in teaching work, makes workers increasingly available to work outside their own environment to perform and carry out their activities, assuming more responsibilities and often giving up moments for leisure time ^3^ . These functions, performed remotely, with emphasis on participation in evaluation boards, the use of applications such as Whatsapp®️ to communicate with students and colleagues from work, as well as participation in virtual meetings, can intensify the overload of teaching work and consequently affect the worker’s health conditions, especially in mental health.

Finally, the design of this study did not allow analyzing the specific causes of the factors associated with both workaholism and burnout. However, this study is a pioneer in investigating workaholism and burnout in professors who work in PPGLL and provides scientific evidence for the construction of knowledge in health, as well as for the elaboration of actions to promote the mental health of these professors.

The results allow managers and professors to reflect on the criteria that guide their work processes, so that they adopt management models, institutional regulatory policies, and appropriate strategies to ensure better working conditions and health for professors.

## CONCLUSION

There was a statistically significant association of excessive work and compulsive work with high emotional exhaustion and high depersonalization in the professionals investigated. Professors with a high level of excessive work had 2.75 times the chance of high emotional exhaustion and 2.08 times the chance of high depersonalization. Regarding professors who showed a high level of compulsive work, they had 4.88 times the chance of emotional exhaustion and 2.97 times the chance of high depersonalization.
