# Supplementary material for: Workaholism and burnout among stricto sensu graduate professors
Source: Rev Saude Publica. 2022 May 18;56:56. doi: 10.11606/s1518-8787.2022056003883 (PMC9165635; doi:10.11606/s1518-8787.2022056003883)
Supplement: Translation

# Translation: Workaholism e burnout entre docentes de pós-graduação stricto sensu

**Authors:** Maynara Fernanda Carvalho Barreto, Maria José Quina Galdino, Frederico Garcia Fernandes, Júlia Trevisan Martins, Maria Helene Palucci Marziale, Maria do Carmo Fernandez Lourenço Haddad

**Affiliations:** I Brasil Universidade Estadual do Norte do Paraná. Setor de Enfermagem. Bandeirantes, PR, Brasil; II Brasil Universidade Estadual de Londrina. Departamento de Letras Vernáculas e Clássicas. Londrina, PR, Brasil; III Brasil Universidade Estadual de Londrina. Departamento de Enfermagem. Londrina, PR, Brasil; IV Brasil Universidade de São Paulo. Escola de Enfermagem de Ribeirão Preto. Ribeirão Preto, SP, Brasil

**Keywords:** Docentes, Esgotamento Profissional, Carga de Trabalho, Estresse Ocupacional, Condições de Trabalho, Saúde Mental

## Abstract

**OBJETIVO:**

Analisar a associação entre trabalho excessivo e trabalho compulsivo com as dimensões da síndrome de *burnout* em docentes de mestrado e doutorado em Letras e Linguística do Brasil.

**MÉTODOS:**

Estudo transversal realizado com 585 docentes permanentes de pós-graduação *stricto sensu* em Letras e Linguística do Brasil. A obtenção dos dados ocorreu entre fevereiro e agosto de 2019, por meio de um questionário online. Os desfechos deste estudo foram as dimensões trabalho compulsivo e trabalho excessivo da escala *Dutch Work Addiction Scale* , as dimensões da *Maslach Burnout Inventory* ^TM^ e seus fatores associados, identificados por modelos múltiplos de regressão logística.

**RESULTADOS:**

Docentes com alto nível de trabalho excessivo (29,40%) apresentaram 2,75 vezes a chance de exaustão emocional alta e 2,08 vezes a chance de despersonalização alta. Quanto aos docentes com alto nível de trabalho compulsivo (8,03%), apresentaram 4,88 vezes a chance de exaustão emocional alta e 2,97 vezes a chance de despersonalização alta. Não foi identificado associação entre trabalho excessivo e trabalho compulsivo com a baixa realização profissional.

**CONCLUSÃO:**

Os resultados mostraram que existe uma associação estatisticamente significativa entre trabalho excessivo e trabalho compulsivo com exaustão emocional alta e despersonalização alta, possibilitando aos gestores e docentes refletirem os critérios que norteiam seus processos laborais, a fim de adotarem modelos de gestão, políticas reguladoras institucionais e estratégias adequadas para melhorar as condições de trabalho e saúde dos docentes.

## INTRODUÇÃO

O *workaholism,* fenômeno crescente de vício ou dependência comportamental no labor, com desenvolvimento progressivo, caracteriza-se pelas dimensões denominadas trabalho excessivo e trabalho compulsivo em trabalhadores a partir da associação de aspectos ocupacionais e individuais ^1^ .

Esse fenômeno está entre as principais causas de influência negativa na vida e adoecimento físico e mental em trabalhadores ^2^ , a partir de uma preocupação excessiva com o trabalho, estimulado por uma motivação incontrolável, maior tempo e esforço investidos em suas atividades laborais ^2 , 3^ . Denomina-se de *workaholic* ou “viciados em trabalho” indivíduos que apresentam comportamento de trabalho excessivo em relação às horas de sua vida e esforço dedicados ao trabalho ^4^ .

Quanto ao desenvolvimento do *workaholism* em docentes universitários, destacam-se os aspectos ocupacionais, em que são necessárias muitas horas de dedicação com intenso esforço mental, físico e emocional para a realização de suas atividades, muitas vezes, caracterizado por uma carga horária excessiva e alterações no comportamento relacionado a uma compulsão por trabalho para conciliar e obter êxito em suas atividades laborais ^5^ .

O *workaholism* também pode prejudicar as relações interpessoais dos trabalhadores, influenciar em suas condições de saúde ^6^ e apresentar-se como um fenômeno correlacionado positivamente com outras doenças ocupacionais como a depressão e a síndrome de *burnout* ^7^ .

O *burnout* ou esgotamento profissional, ocorre a partir de uma resposta prolongada aos fatores interpessoais crônicos e o ambiente de trabalho ^8^ . Estima-se que a síndrome está entre as condições de saúde que acomete um em cada dez trabalhadores em diferentes grupos ocupacionais ^9^ e apresenta alta prevalência entre profissionais da educação, como os docentes universitários ^10^ .

O *burnout* é caracterizado a partir de três dimensões denominadas exaustão emocional, despersonalização e baixa realização profissional ^8^ . A exaustão emocional refere-se à primeira fase e fator principal da doença, correspondendo a uma resposta identificada a partir da redução de recursos físicos e emocionais do trabalhador, caracterizada pelo sentimento de sobrecarga, esgotamento emocional, sensação de fadiga crônica, falta de energia para a realização das atividades do trabalho, irritabilidade excessiva, incapacidade de relaxamento e de recuperação do equilíbrio homeostático ^8^ .

A despersonalização representa os desajustes relacionados ao contexto interpessoal do *burnout* e, quando presente, os trabalhadores se referem a insensibilidade em suas relações interpessoais, desapego excessivo e distanciamento a vários aspectos relacionados ao trabalho, adoção de comportamentos e atitudes insensíveis, impessoais, frieza nos relacionamentos do trabalho e redução no desempenho das atividades ocupacionais ^8^ .

A baixa realização profissional, também denominada ineficácia profissional, corresponde ao componente de autoavaliação do *burnout* , uma vez que o fenômeno inclui variáveis cognitivas-atitudinais como a diminuição da realização pessoal no trabalho ^8^ . Em relação aos aspectos que predizem o *burnout* , destacam-se os fatores individuais, sociais e organizacionais ^11^ .

A partir da definição da síndrome de *burnout* e do *workaholism* e considerando os aspectos ocupacionais e individuais, destaca-se a importância de investigar o trabalho de docentes que atuam em programas de pós-graduação (PPG) nos quais desempenham um papel fundamental e estratégico na produção e consolidação da informação nas áreas de conhecimento científico e tecnológico ^12 , 13^ .

O processo de trabalho do docente de pós-graduação e as suas condições de trabalho podem resultar em insatisfações do professor e em problemas de saúde ^14^ , uma vez que as atividades desempenhadas possuem uma complexidade própria, devido às especificidades do processo laboral do docente como a carga horária elevada ^13^ .

Defende-se a hipótese de que dentre as profissões que estão pré-dispostas a desenvolver *burnout e workaholism* estão os docentes de PPG, visto que há altas exigências para produção científica, atividades fragmentadas como aula em graduação, atividades externas associadas à pós-graduação, dentre outras características ocupacionais que podem potencializar o desenvolvimento desses fenômenos e sua associação.

Portanto, conhecer os aspectos associados ao *workaholism,* ao *burnout* e analisar a associação entre suas dimensões, em docentes que atuam em programas de pós-graduação *stricto sensu* em Letras e Linguística (PPGLL) é fundamental, considerando que no Brasil, estudos sobre o *burnout* em professores, por exemplo, apresentam-se em fase de consolidação ^15^ .

Diante do exposto, este estudo teve como pergunta de pesquisa “Existe associação entre trabalho excessivo e trabalho compulsivo sobre as dimensões da síndrome de *burnout* em docentes de pós-graduação *stricto sensu* em Letras e Linguística do Brasil?”. Para tanto, o objetivo deste estudo foi analisar a associação entre trabalho excessivo e trabalho compulsivo sobre as dimensões da síndrome de *burnout* em docentes de mestrado e doutorado em Letras e Linguística do Brasil.

## MÉTODOS

Estudo transversal realizado com docentes de mestrado e doutorado em Letras e Linguística do Brasil. Foram incluídos docentes com vínculo permanente nos PPGLL, totalizando 585 participantes. Foram excluídos docentes que apresentavam licença de qualquer natureza no ano de 2018.

Para a identificação dos docentes pertencentes aos 155 PPGLL, foi realizado uma busca manual entre os meses de janeiro e abril de 2019 na plataforma Sucupira, ferramenta para coletar informações, realizar análises e avaliações e uma base de referência do Sistema Nacional de Pós-Graduação do Brasil ^16^ . Para a coleta das informações referentes aos e-mails de contato de cada docente, realizou-se a busca manual nas páginas eletrônicas de cada programa e foram encaminhados e-mails aos coordenadores dos cursos, solicitando as informações que não estavam disponíveis em suas páginas, de forma a complementar as disponíveis nas páginas eletrônicas, tendo sido identificado 2.752 docentes credenciados aos PPGLL.

Quanto ao cálculo do poder amostral, utilizou-se a fórmula n ≥ 50 +8m (m: número de variáveis independentes) para testar múltiplas associações ^17^ . Assim, neste estudo foram considerados 38 variáveis independentes mais as duas dimensões do *workaholism* , o que indicou o mínimo de 370 participantes (n ≥ 50 + (8 x 40)).

A coleta de dados ocorreu entre fevereiro e agosto de 2019. Para essa etapa, foi desenvolvida uma plataforma virtual, denominada *Mubble,* contendo um questionário semiestruturado com variáveis sociodemográficas, de saúde, hábitos de vida e ocupacionais, em que todas as questões possuíam resposta obrigatória.

A síndrome de *burnout* , desfecho do estudo, foi avaliada pelo *Maslach Burnout Inventory* ^TM^ *– Human Services Survey* (MBI ^TM^ -HSS) em sua versão traduzida para o português ^18^ e validada para uso no Brasil ^19^ , constituída por 22 itens distribuídos em suas três dimensões: exaustão emocional (nove itens), despersonalização (cinco itens) e baixa realização profissional (oito itens). As respostas podem ser mensuradas em uma escala do tipo Likert de sete pontos, variando de zero a seis.

Para a análise das respostas obtidas, as três dimensões devem ser analisadas e igualmente consideradas como um construto, com o objetivo de considerar a síndrome em potencialidade de desenvolvimento e não como um diagnóstico ^20^ . Dessa forma, quando um indivíduo apresenta simultaneamente altas pontuações em exaustão emocional e despersonalização e baixas pontuações em realização profissional, pode apresentar chances maiores para o desenvolvimento da síndrome de *burnout* ^21^ .

Em relação ao *workaholism* , utilizou-se a *Dutch Work Addiction Scale* (DUWAS) em suas duas dimensões denominadas de trabalho excessivo e trabalho compulsivo, traduzida e validada para o português ^22^ . Para tanto, os dados foram dicotomizados em alto ou baixo a partir do percentil 75.

A partir do banco de dados realizou-se as análises estatísticas com as seguintes variáveis: sexo (masculino ou feminino), faixa etária (29–49 anos ou 50–78 anos), localização geográfica da universidade (Centro-Oeste, Norte, Nordeste, Sul ou Sudeste), qualidade de vida (ruim ou boa), oportunidades de lazer (poucas ou muitas), padrão de sono (ruim ou bom), ativo fisicamente (não ou sim), tipo de instituição (pública ou privada), dois vínculos em PPGLL (não ou sim), orienta mestrado e doutorado (não ou sim), tempo de atuação em PPGLL (≤ 9 anos ou ≥ 10 anos), número de orientandos (≤ 5 alunos ou ≥ 6 alunos), conceito Capes (3–5 ou 6–7).

Para a análise dos dados, utilizou-se o *Statistical Package of Social Sciences* (SPSS), versão 20.0 (IBM, Chicago, IL, USA). O desfecho deste estudo foram as dimensões exaustão emocional, despersonalização e baixa realização profissional do MBI ^TM^ -HSS e as dimensões trabalho excessivo e trabalho compulsivo do DUWAS, realizando análise bivariada e o cálculo das frequências absolutas e relativas.

A associação entre a síndrome de *burnout* (variável dependente), trabalho excessivo e trabalho compulsivo (variáveis independentes) foi verificada por meio de modelos múltiplos de regressão logística considerando as variáveis de ajuste: sexo, faixa etária, ser professor sênior e anos de atuação no mestrado e doutorado. Os resultados foram apresentados em *odds ratio* brutos e ajustados com intervalos de confiança de 95%.

O desenvolvimento do estudo atendeu às normas de ética em pesquisa nacionais e internacionais, incluindo aprovação conforme Parecer n°. 2.347.839, CAAE: 79006017.0.0000.5231.

## RESULTADOS

Dos 585 participantes, a maioria era mulher (372; 63,59%), sendo que 298 (50,94%) docentes apresentavam idade entre 29 e 49 anos. Quanto às condições de saúde, 423 (72,31%) não eram ativos fisicamente, 434 (74,19%) consideravam sua qualidade de vida boa. No entanto, 341 (58,29%) responderam possuir padrão de sono ruim e 459 (78,46%) mencionaram não possuir oportunidade para lazer.

Quanto à participação geográfica dos PPGLL, 204 (34,87%) docentes eram vinculados a programas da região Sudeste, 165 (28,21%) da região Sul, 121 (20,68%) da região Nordeste, 62 (10,60%) da região Centro-Oeste, e 33 (5,64%) de programas da região Norte.

A alta exaustão emocional associou-se à faixa etária, qualidade de vida, oportunidades de lazer, padrão de sono, ser ativo fisicamente e tempo de atuação nos PPGLL. A alta despersonalização associou-se à faixa etária, localização geográfica da universidade, qualidade de vida, padrão de sono, tempo de atuação nos PPGLL e conceito Capes. A baixa realização profissional associou-se à faixa etária, localização geográfica da universidade, qualidade de vida, oportunidades de lazer, padrão de sono, tempo de atuação nos PPGLL, e número de orientandos ( Tabela 1 ).

**Tabela 1:** Associação das variáveis sociodemográficas, ocupacionais e de hábitos de vida com as dimensões da síndrome de *burnout* em docentes de mestrado e doutorado em Letras e Linguística do Brasil, 2020.

Variável de análise	Exaustão emocional alta		p	Despersonalização alta		p	Realização profissional baixa		p
	Não	Sim		Não	Sim		Não	Sim	
	n (%)	n (%)		n (%)	n (%)		n (%)	n (%)	
Sexo									
Masculino	112 (52,6)	101 (47,4)	0,77	180 (84,5)	33 (15,5)	0,39	151 (70,9)	62 (29,1)	0,15
Feminino	191 (51,3)	181 (48,7)		324 (87,1)	48 (12,9)		284 (76,3)	88 (23,7)	
Faixa etária									
29–49 anos	126 (42,3)	172 (57,7)	**< 0,01**	244 (81,9)	54 (18,1)	**< 0,01**	209 (70,1)	89 (29,9)	**< 0,01**
50–78 anos	177 (61,7)	110 (38,3)		260 (90,6)	27 (9,4)		226 (78,7)	61 (21,3)	
Localização geográfica da universidade									
Centro-Oeste	31 (50)	31 (50)	0,19	50 (80,6)	12 (19,4)	**< 0,01**	51 (82,3)	11 (17,7)	**< 0,01**
Norte	13 (39,4)	20 (60,6)		24 (72,7)	9 (27,3)		20 (60,6)	13 (39,4)	
Nordeste	73 (60,3)	48 (39,7)		109 (90,1)	12 (9,9)		101 (83,5)	20 (16,5)	
Sul	82 (49,7)	83 (50,3)		138 (83,6)	27 (16,4)		111 (67,3)	54 (32,7)	
Sudeste	104 (51)	100 (49)		183 (89,7)	21 (10,3)		152 (74,5)	52 (25,5)	
Qualidade de vida									
Ruim	39 (25,8)	112 (74,2)	**< 0,01**	121 (80,1)	30 (19,9)	**< 0,01**	92 (60,9)	59 (39,1)	**< 0,01**
Boa	264 (60,8)	170 (39,2)		383 (88,2)	51 (11,8)		343 (79)	91 (21)	
Oportunidades de lazer									
Poucas	207 (45,1)	252 (54,9)	**< 0,01**	390 (85)	69 (15)	0,10	327 (71,2)	132 (28,8)	**< 0,01**
Muitas	96 (76,2)	30 (23,8)		114 (90,5)	12 (9,5)		108 (85,7)	18 (14,3)	
Padrão de sono									
Ruim	135 (39,6)	206 (60,4)	**< 0,01**	283 (83)	58 (17)	**< 0,01**	235 (68,9)	106 (31,1)	**< 0,01**
Bom	168 (68,9)	76 (31,1)		221 (90,6)	23 (9,4)		200 (82)	44 (18)	
Ativo fisicamente									
Não	201 (47,5)	222 (52,5)	**< 0,01**	359 (84,9)	64 (15,1)	0,13	307 (72,6)	116 (27,4)	0,11
Sim	102 (63)	60 (37)		145 (89,5)	17 (10,5)		128 (79)	34 (21)	
Tipo de instituição									
Pública	265 (51,4)	251 (48,6)	0,56	445 (86,2)	71 (13,8)	0,87	383 (74,2)	133 (25,8)	0,84
Privada	38 (55,1)	31 (44,9)		59 (85,5)	10 (14,5)		52 (75,4)	17 (24,6)	
Dois vínculos em PPGLL									
Não	235 (50,5)	230 (49,5)	0,23	403 (86,7)	62 (13,3)	0,49	342 (73,5)	123 (26,5)	0,37
Sim	68 (56,7)	52 (43,3)		101 (84,2)	19 (15,8)		93 (77,5)	27 (22,5)	
Orienta mestrado e doutorado									
Não	88 (47,6)	97 (52,4)	0,16	152 (82,2)	33 (17,8)	0,06	128 (69,2)	57 (30,8)	0,05
Sim	215 (53,8)	185 (46,3)		352 (88)	48 (12)		307 (76,8)	93 (23,3)	
Tempo de atuação nos PPGLL									
≤ 9 anos	153 (45,7)	182 (54,3)	**< 0,01**	278 (83)	57 (17)	**< 0,01**	232 (69,3)	103 (30,7)	**< 0,01**
≥ 10 anos	150 (60)	100 (40)		226 (90,4)	24 (9,6)		203 (81,2)	47 (18,8)	
Número de orientandos									
≤ 5 alunos	160 (50,5)	157 (49,5)	0,49	272 (85,8)	45 (14,2)	0,79	224 (70,7)	93 (29,3)	**< 0,01**
≥ 6 alunos	143 (53,4)	125 (46,6)		232 (86,6)	36 (13,4)		211 (78,7)	57 (21,3)	
Conceito Capes									
3–5	222 (51,3)	211 (48,7)	0,67	364 (84,1)	69 (15,9)	**< 0,01**	321 (74,1)	112 (25,9)	0,83
6–7	81 (53,3)	71 (47,7)		140 (92,1)	12 (7,9)		114 (75)	38 (25)	

PPGLL: programa de pós-graduação *stricto sensu* em Letras e Linguística.

O trabalho excessivo em nível elevado associou-se à faixa etária, qualidade de vida, oportunidades de lazer, padrão de sono, ser ativo fisicamente e ao tempo de atuação nos PPGLL. Já o trabalho em nível compulsivo alto associou-se às variáveis qualidade de vida, oportunidades de lazer, padrão de sono e ser ativo fisicamente ( Tabela 2 ).

**Tabela 2:** Associação das variáveis sociodemográficas, ocupacionais e de hábitos de vida com o trabalho excessivo e compulsivo em docentes de mestrado e doutorado em Letras e Linguística do Brasil. Brasil, 2020.

Variável de análise	Nível excessivo		p	Nível compulsivo		p
	Baixo	Alto		Baixo	Alto	
	n (%)	n (%)		n (%)	n (%)	
Sexo						
Masculino	157 (73,7)	56 (26,3)	0,21	200 (93,9)	13 (6,1)	0,19
Feminino	256 (68,8)	116 (31,2)		338 (90,9)	34 (9,1)	
Faixa etária						
29–49 anos	190 (63,8)	108 (36,2)	**< 0,01**	272 (91,3)	26 (8,7)	0,53
50–78 anos	223 (77,7)	64 (22,3)		266 (92,7)	21 (7,3)	
Localização geográfica da universidade						
Centro-Oeste	40 (64,5)	22 (35,5)	0,18	56 (90,3)	6 (9,7)	0,96
Norte	22 (66,7)	11 (33,3)		32 (97)	1 (3)	
Nordeste	89 (73,6)	32 (26,4)		111 (91,7)	10 (8,3)	
Sul	108 (65,5)	57 (34,5)		151 (91,5)	14 (8,5)	
Sudeste	154 (75,5)	50 (24,5)		188 (92,2)	16 (7,8)	
Qualidade de vida						
Ruim	84 (55,6)	67 (44,4)	**< 0,01**	124 (82,1)	27 (17,9)	**< 0,01**
Boa	329 (75,8)	105 (24,2)		414 (95,4)	20 (4,6)	
Oportunidades de lazer						
Poucas	308 (67,1)	151 (32,9)	**< 0,01**	414 (90,2)	45 (9,8)	**< 0,01**
Muitas	105 (83,3)	21 (16,7)		124 (98,4)	2 (1,6)	
Padrão de sono						
Ruim	214 (62,8)	127 (37,2)	**< 0,01**	301 (88,3)	40 (11,7)	**< 0,01**
Bom	199 (81,6)	45 (18,4)		237 (97,1)	7 (2,9)	
Ativo fisicamente						
Não	284 (67,1)	139 (32,9)	**< 0,01**	383 (90,5)	40 (9,5)	**< 0,01**
Sim	129 (79,6)	33 (20,4)		155 (95,7)	7 (4,3)	
Tipo de instituição						
Pública	362 (70,2)	154 (29,8)	0,52	475 (92,1)	41 (7,9)	0,83
Privada	51 (73,9)	18 (26,1)		63 (91,3)	6 (8,7)	
Dois vínculos em PPGLL						
Não	325 (69,9)	140 (30,1)	0,46	429 (92,3)	36 (7,7)	0,61
Sim	88 (73,3)	32 (26,7)		109 (90,8)	11 (9,2)	
Orienta mestrado e doutorado						
Não	126 (68,1)	59 (31,9)	0,37	172 (93)	13 (7)	0,54
Sim	287 (71,8)	113 (28,2)		366 (91,5)	34 (8,5)	
Tempo de atuação nos PPGLL						
≤ 9 anos	221 (66)	114 (34)	**< 0,01**	307 (91,6)	28 (8,4)	0,73
≥ 10 anos	192 (76,8)	58 (23,2)		231 (92,4)	19 (7,6)	
Número de orientandos						
≤ 5 alunos	222 (70)	95 (30)	0,74	292 (92,1)	25 (7,9)	0,89
≥ 6 alunos	191 (71,3)	77 (28,7)		246 (91,8)	22 (8,2)	
Conceito Capes						
3–5	302 (69,7)	131 (30,3)	0,44	399 (92,1)	34 (7,9)	0,79
6–7	111 (73)	41 (27)		139 (91,4)	13 (8,6)	

PPGLL: programa de pós-graduação *stricto sensu* em Letras e Linguística.

A Tabela 3 mostra que docentes com alto nível de trabalho excessivo apresentaram 2,75 vezes a chance de exaustão emocional alta e 2,08 vezes a chance de despersonalização alta. Os docentes que apresentaram alto nível de trabalho compulsivo apresentaram 4,88 vezes a chance de exaustão emocional e 2,97 vezes a chance de despersonalização alta.

**Tabela 3:** Associação entre as dimensões da síndrome de *burnout* e *workaholism* em docentes de mestrado e doutorado em Letras e Linguística do Brasil. Brasil, 2020.

Modelo Múltiplo	p	Odds ratio ^Bruto^ (IC95%)	p	Odds ratio ^Ajustado^ (IC95%)
Exaustão emocional alta				
Trabalho excessivo	**< 0,01**	2.082 (2.119–4.483)	**< 0,01**	2.754 (1.876–4.043)
Trabalho compulsivo	**< 0,01**	5.087 (2.412–10.729)	**< 0,01**	4.876 (2.291–10.377)
Despersonalização alta				
Trabalho excessivo	**< 0,01**	2.298 (1.423–3.713)	**< 0,01**	2.076 (1.272–3.387)
Trabalho compulsivo	**< 0,01**	2.982 (1.518–5.860)	**< 0,01**	2.972 (1.495–5.907)
Baixa realização profissional				
Trabalho excessivo	0,07	1.451 (0.977–2.155)	0,14	1.356 (0.904–2.034)
Trabalho compulsivo	0,74	1.119 (0.574–2.183)	0,73	1.127 (0.573–2.218)

IC95%: intervalo de confiança de 95%.

Nota: ajustado por sexo, faixa etária, professor sênior e anos de atuação no mestrado e doutorado. Ajustado por sexo, faixa etária, professor sênior e anos de atuação no mestrado e doutorado.

## DISCUSSÃO

A análise da associação entre trabalho excessivo e trabalho compulsivo com as dimensões exaustão emocional, despersonalização e baixa realização profissional da síndrome de *burnout* mostrou que os docentes com altos níveis de trabalho excessivo e trabalho compulsivo apresentaram chances aumentadas de exaustão emocional e despersonalização. Observou-se que o trabalho excessivo e trabalho compulsivo não foram significativamente associados à baixa realização profissional.

Estudo de Clark et al. ^23^ mostrou por meio de metanálise uma relação positiva entre *workaholism* e *burnout* . Os resultados do estudo apresentaram correlações maiores com as dimensões de exaustão emocional (p = 0,42) e despersonalização (p = 0,29) em relação à baixa realização profissional. Outro estudo ^4^ , realizado com o objetivo de comparar o *workaholism* como preditor para o *burnout* em grupos ocupacionais da China e dos Estados Unidos, mostrou o *workaholism* positivamente correlacionado a exaustão emocional e despersonalização. Os resultados de ambas investigações corroboram os achados do presente estudo.

Em relação ao trabalho docente, sobretudo em cursos de mestrado e doutorado, considera-se que a intensificação das atividades laborais desenvolvidas pode ser um fator que potencializa o trabalho excessivo, o trabalho compulsivo e, consequentemente, a exaustão emocional e despersonalização. Esse fator refere-se ao aumento da carga de trabalho esperada de um trabalhador, elevando o número de tarefas a serem realizadas ou diminuindo o tempo necessário para concluir as demandas exigidas ^4^ .

Pressupõe-se que o trabalho docente resulta em um intenso desgaste cognitivo em que os professores enfrentam mais de 40 horas de trabalho por semana, além de disporem de tempo para participar de atividades extraclasse, atividades burocráticas e estarem inseridos no desenvolvimento de pesquisas. Assim, com um aumento de funções e atividades delegadas, trabalhadores passam mais horas para cumprir os seus prazos e funções de modo que mais horas de trabalho podem levar a maior fadiga, estresse no trabalho e desequilíbrio na vida profissional ^4^ .

Importante estudo ^24^ realizado com docentes universitários de 13 países mostrou que já em 2012 a média de horas trabalhadas por seus participantes foi de 48,4 horas por semana, e aproximadamente 20% deles relataram trabalhar mais de 60 horas entre segunda e sexta-feira.

Em relação às variáveis que podem ser associadas tanto ao *workaholism* como ao *burnout* em docentes universitários, sobretudo os que atuam em cursos de mestrado e doutorado, observa-se que a pressão para o desenvolvimento de pesquisas e publicação científica são os principais fatores de estresse ocupacional ^14^ e que podem estar associados a altos níveis de exaustão emocional e despersonalização ^25^ .

Destaca-se que a exaustão emocional aumenta conforme o trabalhador possui maior percepção de que a profissão é estressante, acredita que a profissão está interferindo na vida pessoal e/ou considera suas atividades de trabalho menos interessantes do que quando começou. Já em relação à despersonalização, seus níveis podem aumentar com maior tempo de serviço, percepção das expectativas familiares, mau comportamento dos alunos, falta de autonomia, estabilidade e trabalho como espaço total de vida e menor satisfação com o crescimento profissional ^16^ .

Quanto aos fatores associados às dimensões do *workaholism* e *burnout* deste estudo, destaca-se que a porcentagem de docentes de até 49 anos com exaustão emocional alta, despersonalização alta, realização profissional baixa e nível de trabalho excessivo alto foi maior do que entre os docentes com mais de 50 anos de idade. Esses achados corroboram a afirmativa de que o *workaholism* pode estar significativamente correlacionado com a faixa etária na qual indivíduos mais novos apresentam altos níveis de trabalho excessivo e trabalho compulsivo em relação aos de idade mais elevada ^26^ .

Destaca-se que o *burnout* é comum em professores mais jovens ^27^ . Já em relação às suas dimensões, a exaustão emocional diminui de acordo com a elevação da idade e a despersonalização pode elevar com o aumento da idade, sendo mais comum na faixa etária de 40 a 59 anos. No entanto, resultados relacionados à relação entre essas variáveis, em professores, ainda são incipientes ^15^ , sobretudo em docentes que atuam em PPG.

Observou-se que na localização geográfica houve diferença significativa entre a região da universidade em que os participantes atuavam para a despersonalização e realização profissional baixa. Isso pode estar relacionado à desigualdade na construção e distribuição regional dos PPG no Brasil, em que se evidencia uma forte concentração nas regiões mais ricas do país ^13^ e favorecimento das regiões Sudeste, Sul e o Distrito Federal, fundamentalmente para os estados do Rio de Janeiro e São Paulo ^28^ .

Defende-se a hipótese de que a assimetria e desigualdades oriundas da construção da pós-graduação *stricto sensu* no Brasil apresentam uma influência no perfil dos PPGLL, exigências dos programas no desenvolvimento do trabalho docente, forma como os docentes se sentem e desempenham suas funções e, consequentemente nas condições de trabalho e saúde física e mental dos professores.

Os docentes do presente estudo que referiram possuir mais de dez anos de atuação em PPGLL apresentaram porcentagens menores de trabalho excessivo alto, exaustão emocional alta, despersonalização alta e realização profissional baixa, quando comparados aos docentes com menos de nove anos de atuação. No entanto, a porcentagem de realização profissional baixa foi menor entre docentes que afirmaram possuir mais de seis orientandos.

Destaca-se que não foram identificados estudos que pudessem amparar a discussão desses resultados, porém, supõe-se que os docentes que possuem mais de seis orientandos possuem um maior tempo de docência, apresentam suas carreiras consolidadas e uma maior estratégia de enfrentamento em relação aos aspectos ocupacionais dos seus processos de trabalho, contribuindo para maior satisfação profissional e prazer com o trabalho.

Docentes pertencentes a cursos com conceito Capes seis e sete apresentaram porcentagem menor de despersonalização alta em relação aos cursos com notas entre três e cinco. Embora as características do trabalho sejam diferentes em instituições privadas e públicas ^14^ , as exigências de avaliação em relação aos PPG pela Coordenação de Aperfeiçoamento de Pessoal de Nível Superior (Capes), órgão governamental regulador da pós-graduação no Brasil, são únicas para a área.

No entanto, presume-se que os PPGLL com conceitos de três a cinco estão em busca da consolidação e excelência, bem como do reconhecimento de seus programas, manutenção e ampliação dos recursos para os cursos e, a partir das exigências apresentadas aos docentes e do modelo de avaliação adotado, em longo prazo, podem ser fatores associados à despersonalização desses profissionais.

Sobre o modelo de avaliação dos PPG *stricto sensu* realizado pela Capes, a competição que emerge a partir das exigências cobradas para o desempenho dos cursos de mestrado e doutorado, permanência ou desligamento desses programas, são fatores de questionamento por parte dos docentes em relação ao seu sentimento de pertencimento aos programas. No caso da área de Letras e Linguística, apesar de existir métricas para avaliação da área, somam-se ainda a exclusão de temas das humanidades nas políticas públicas de financiamento e sua constituição histórica marcada pela escassez de apoio por agências de fomento, recebimento de menores recursos e incentivo à pesquisa quando comparada às áreas que constituem as Ciências Humanas e a necessidade de combater o produtivismo na pós-graduação e seus efeitos sobre a política científica brasileira ^29^ .

Ainda sobre o produtivismo, a cobrança por produtividade e obtenção de recurso financeiro para pesquisa associa-se a um processo cíclico em que a percepção subjetiva de pressão por publicações e produtos pode implicar em aumento do ritmo e carga de trabalho e, como consequência, em comprometimento excessivo, estresse e maior esforço para o desenvolvimento das atividades laborais ^14^ .

Os docentes do presente estudo que afirmaram ter boa qualidade de vida apresentaram uma porcentagem menor de altos níveis de trabalho excessivo e trabalho compulsivo, exaustão emocional alta, despersonalização alta e realização profissional baixa, quando comparados aos docentes que apontaram ter qualidade de vida ruim.

A qualidade de vida de um indivíduo constitui-se como um importante fator de saúde que pode ser afetado pelo *workaholism* , pois, a partir do comportamento de trabalho excessivo e compulsivo, muitas vezes a fadiga do trabalhador não é reconhecida ^3^ . Independentemente da cultura, o *workaholism* é prejudicial ao bem-estar dos trabalhadores por associar-se a altos níveis de tensão no trabalho e, consequentemente, altos níveis de exaustão emocional, baixa qualidade de vida e qualidade de sono comprometida ^26^ . Assim, tanto os indivíduos como as organizações de trabalho necessitam de estratégias de mudança ^4^ .

Neste estudo, os participantes que referiram possuir um padrão de sono bom apresentaram uma porcentagem menor de altos níveis de trabalho excessivo e trabalho compulsivo, alta exaustão emocional, despersonalização alta e realização profissional baixa, quando comparados aos docentes que referiram possuir padrão de sono ruim. Acrescenta-se também que os docentes do presente estudo que referiram ser ativos fisicamente apresentaram uma porcentagem menor de trabalho excessivo alto, trabalho compulsivo alto e exaustão emocional alta. A realização de atividade física e o sono são importantes para a recuperação do ser humano minimizando um processo de irritação ou extenuação no corpo e mente ^5^ .

Quanto à atividade física, a Organização Mundial da Saúde recomenda que as pessoas entre 18 e 64 anos pratiquem semanalmente pelo menos 150 minutos de atividade leve ou moderada ou, no mínimo, 75 minutos de atividade de maior intensidade ^30^ . Essa prática é considerada um fator de proteção para o *burnout* em docentes universitários ^10^ . No entanto, estudo ^5^ realizado com 270 docentes universitários mostrou a falta de atividade física no tempo livre por quase um terço (29,6%) dos participantes, semelhante aos achados neste estudo.

Quanto ao sono, é considerado um importante fator de proteção, restauração física e equilíbrio de saúde para o ser humano desde que seja suficiente e de qualidade. No entanto, desequilíbrios nesse fator podem afetar a performance cognitiva, mental e física do indivíduo e é considerado um sério problema na manutenção da saúde e de trabalhadores, sobretudo os *workaholics* ^26^ .

Desequilíbrios no padrão e na qualidade do sono são considerados fatores que constituem um dos resultados do trabalhador que apresenta vício de trabalho em diferentes grupos ocupacionais, resultando em menor qualidade de vida ^2 , 26^ . Docentes universitários apresentam uma associação significativa entre a sonolência diurna excessiva e menores escores em itens como capacidade funcional, aspectos físicos, dor, estado geral da saúde, vitalidade, aspectos sociais, aspecto emocional e saúde emocional ^5^ .

Os docentes do presente estudo que referiram possuir muitas oportunidades de lazer apresentaram uma porcentagem menor de altos níveis de trabalho excessivo e trabalho compulsivo, alta exaustão emocional e realização profissional baixa, quando comparados aos docentes que mencionaram possuir poucas oportunidades de lazer. Nessa perspectiva, trabalhadores com altos níveis de trabalho excessivo e trabalho compulsivo devem investir seus recursos cognitivos, emocionais e físicos no processo de trabalho, mas nem sempre conseguem reabastecer seus recursos por meio de atividades restauradoras como o lazer ^4^ .

Ademais, o uso de tecnologias e meios de comunicação disponíveis 24 horas por dia, comumente utilizados no trabalho docente, faz com que os trabalhadores estejam cada vez mais disponíveis para o trabalho fora do ambiente próprio para executar e desempenhar suas atividades, assumindo mais responsabilidades e, muitas vezes, abdicando de momentos para o tempo de lazer ^3^ . Essas funções desempenhadas via remota, com destaque para a participação em bancas de avaliação, o uso de aplicativos como o Whatsapp®️ para comunicação com alunos e colegas de trabalho, bem como a participação em reuniões virtuais podem intensificar a sobrecarga de trabalho docente e consequentemente influenciar nas condições de saúde do trabalhador, sobretudo na mental.

Para finalizar, o delineamento deste estudo não permitiu analisar as causas específicas dos fatores associados tanto do *workaholism* como para o *burnout* . No entanto, o estudo é pioneiro na investigação do *workaholism* e *burnout* em docentes que atuam em PPGLL e fornece evidências científicas para a construção do conhecimento em saúde, bem como para a elaboração de ações para a promoção da saúde mental desses docentes.

Os resultados encontrados permitem aos gestores e docentes refletirem os critérios que norteiam seus processos de trabalho, para que adotem modelos de gestão, políticas reguladoras institucionais e estratégias adequadas para garantirem melhores condições de trabalho e saúde dos docentes.

## CONCLUSÃO

Houve uma associação estatisticamente significativa entre trabalho excessivo e trabalho compulsivo com exaustão emocional alta e despersonalização alta nos profissionais investigados. Docentes com alto nível de trabalho excessivo apresentaram 2,75 vezes a chance de exaustão emocional alta e 2,08 vezes a chance de despersonalização alta. Quanto aos docentes que apresentaram alto nível de trabalho compulsivo, esses apresentaram 4,88 vezes a chance de exaustão emocional e 2,97 vezes a chance de despersonalização alta.
